# Radiobiological Framework for the Evaluation of Stereotactic Radiosurgery Plans for Invasive Brain Tumours

**DOI:** 10.1155/2013/527251

**Published:** 2013-12-29

**Authors:** Helena Sandström, Alexandru Dasu, Iuliana Toma-Dasu

**Affiliations:** ^1^Medical Radiation Physics, Stockholm University and Karolinska Institutet, 171 76 Stockholm, Sweden; ^2^Department of Radiation Physics UHL, County Council of Östergötland, Linköping University, 581 85 Linköping, Sweden

## Abstract

This study presents a radiobiological formalism for the evaluation of the treatment plans with respect to the probability of controlling tumours treated with stereotactic radiosurgery accounting for possible infiltrations of malignant cells beyond the margins of the delineated target. Treatments plans devised for three anaplastic astrocytoma cases were assumed for this study representing cases with different difficulties for target coverage. Several scenarios were considered regarding the infiltration patterns. Tumour response was described in terms of tumour control probability (TCP) assuming a Poisson model taking into account the initial number of clonogenic cells and the cell survival. The results showed the strong impact of the pattern of infiltration of tumour clonogens outside the delineated target on the outcome of the treatment. The treatment plan has to take into account the existence of the possible microscopic disease around the visible lesion; otherwise the high gradients around the target effectively prevent the sterilisation of the microscopic spread leading to low probability of control, in spite of the high dose delivered to the target. From this perspective, the proposed framework offers a further criterion for the evaluation of stereotactic radiosurgery plans taking into account the possible infiltration of tumour cells around the visible target.

## 1. Introduction

The aim of radiation therapy is to stop the tumour growth process with sparing of the normal tissues nearby. For stereotactic radiosurgery (SRS) this is achieved by delivering a highly conformal dose distribution to the target in one fraction. The relatively steep dose falloff around the target ensures the sparing of the normal tissue and/or the critical structures near the target and this is the core of the SRS concept. The evaluation of plans is currently performed as a function of the conformity of therapeutic isodoses to the defined target and the gradients outside the target. This approach intrinsically assumes that tumour cells are confined to the target volume and that there are no infiltrations in the normal tissues around this target or that the impact of the possible infiltrations outside the delineated target on the probability of eradication of the tumour is negligible. However, several of the brain tumours commonly treated with SRS are invasive and therefore the existence of tumour cells outside the tumour lesions that could be identified in diagnostic images cannot be excluded [1, 2].

From this perspective, the evaluation of the plans should be performed not only from purely geometrical and physical points of view, but also from a radiobiological perspective taking into account the invasiveness of the tumours that have to be treated and the distribution of tumour cells in and around the target. Therefore it is the aim of this paper to introduce a radiobiological formalism for the evaluation of the treatment plans with respect to the probability of controlling tumours treated with SRS.

## 2. Materials and Methods

### 2.1. Patient Material and Target Definition

**Figure 1:**
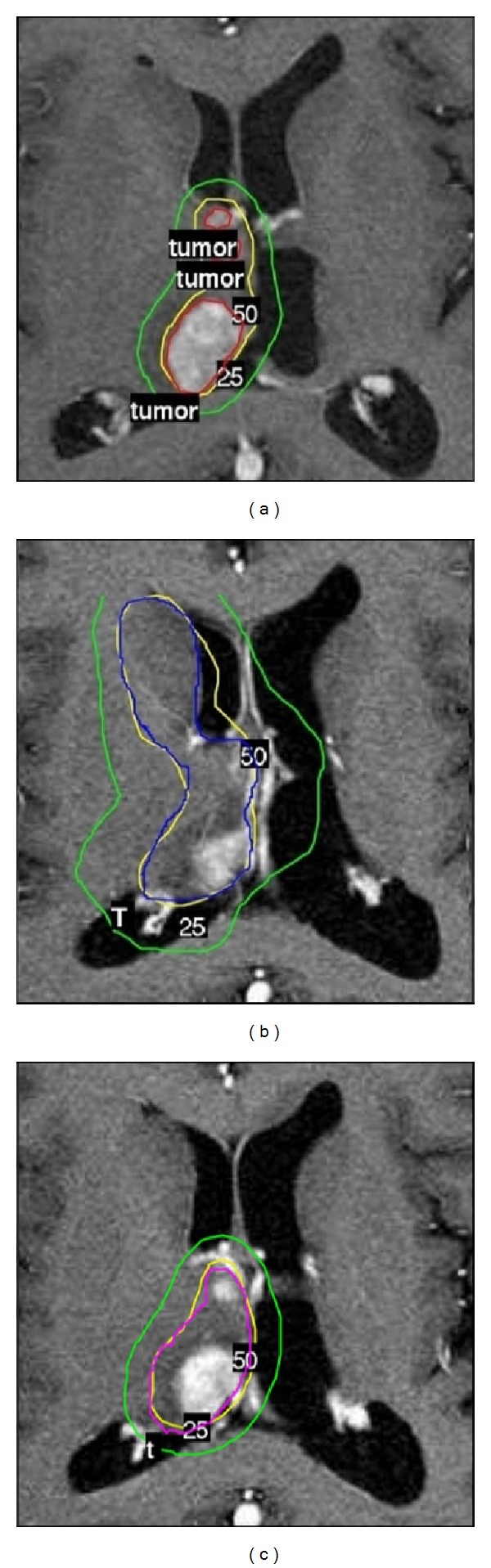
Anaplastic astrocytoma cases considered for this study and the 50% and the 25% isodoses in the treatment plan. CI = 0.77 (case 1, (a)), CI = 0.96 (case 2, (b)) and CI = 0.91 (case 3, (c)).

Three representative cases of recurrent anaplastic astrocytoma have been selected from a series of cases treated with Leksell Gamma Knife Perfexion (Figure 1). The treatment plans were calculated for a prescribed dose to the target of 16 Gy at the 50% isodose. The dose distributions were exported from the treatment planning system and used for calculations together with the structures. Dose matrices were exported from the treatment plans with the same transversal resolution as the structure matrices. The interslice resolution of the structure matrices is given by the imaging method used in each case. All dose matrices were redefined to have the same number of slices and interslice resolution as the structure matrices.

The three panels in Figure 1 illustrate the cases chosen for this study. Case 1 (Figure 1(a)) shows a plan for the anaplastic astrocytoma with poor conformity. The conformity was quantified and expressed as conformity index, defined as the ratio of the volume of the target covered by the prescribed isodose volume and the total target volume [3]. The conformity index (CI) for case 1 was 0.77. A plan for which a much higher CI has been achieved is presented in Figure 1(b), hereby described as case 2. The CI for case 2 was 0.96.  Figure 1(c) illustrates an intermediate situation, case 3, for which the plan leads to a CI of 0.91.

Each of the cases in Figure 1 is not only showing a different coverage of the target but also a different irradiation of the tissue nearby the target.

**Figure 2:**
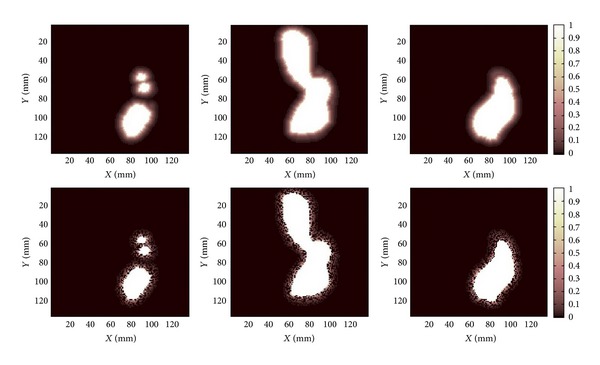
Delineated targets from Figure 1 and patterns of infiltration assumed in this study. Cell density outside the target is assumed to decrease as function of the distance to the target (upper panels) or have a more heterogeneous pattern of infiltration (lower panels). The colour scale indicates the relative density of clonogens, from 1 inside the target volume to zero outside the target if the distance from the border of the target exceeded 10 mm.

In order to account for the invasiveness of the anaplastic astrocytoma clonogenic cells outside the planned target volume, several scenarios regarding infiltration of tumour cells have been considered. Thus, in one scenario it has been assumed that there is no infiltration around the delineated tumour volume and that the cell density is the same in all the voxels of the target. The other two scenarios assumed that tumour clonogenic cells exist outside the delineated area, either as a continuously decreasing function of the distance to the target or assuming a more heterogeneous pattern of infiltration outside the target (Figure 2). Although there are several modelling studies of the tumour margin diffusion and infiltration [4], the information regarding the infiltration pattern of the astrocytoma cells is rather scarce. In absence of histologically validated models for the infiltrations, a simple continuous function describing the decrease of the density of clonogenic cells outside the target with distance as *f*(*d*) = exp⁡(−*d*) was assumed. For modelling the stochastic character of invasiveness suggested by some studies [4], a second scenario was also assumed in which the continuous function describing the decrease of the density of clonogenic cells outside the target with distance was coupled with a random distribution of the clonogens in the voxels outside the target.

In both scenarios, as astrocytomas are highly infiltrative types of gliomas, similar to glioblastomas, the maximal distance for infiltration was based on a study by Yamahara et al. [5] comparing examined autopsy brains and MR images for glioblastoma in which a peripheral tumour boundary infiltration of 6–14 mm was found. Thus, the maximum distance at which the astrocytoma cells could be found outside the borders of the target was considered to be 10 mm, the average value in the study by Yamahara et al. [5].

### 2.2. Radiobiological Model

In order to assess the influence of the tumour cells invasiveness outside the target on the treatment outcome, the response of the tumour to radiosurgery was described in terms of the tumour control probability (TCP) assuming a Poisson model taking into account the initial number of clonogenic cells and the cell survival. Given a dose distribution with doses *D*_*i*_ to voxels *i* in the patient, the tumour control probability is described by

(1) $$\begin{array}{c} \text{TCP}=\exp\left(-\overset{n}{\underset{i=1}{\sum }}\rho_{0}\left(V_{i}\right)V_{i}\;e^{-\alpha D_{i}{-\beta D}_{i}^{2}}\right), \end{array}$$

where *n* is the total number of voxels, *ρ*_0_(*V*_*i*_) is the initial density of clonogenic cells in voxel *i*, *V*_*i*_ is the volume of voxel *i*, and *D*_*i*_ is the dose delivered to the cells in voxel *i*. Equation (1) assumes the LQ model for cell survival [6] with parameters *α* and *β*. It has to be mentioned that the general expression in (1) could be used for response calculation irrespective of the size or shape of the tumour and any given distribution of doses *D*_*i*_.

The radiobiological parameters used in the calculations were *α* = 0.24 Gy^−1^, *β* = 0.03 Gy^−2^, and *α*/*β* = 8.31 Gy as reported in the review by Malaise et al. [7] for glioblastoma. It was further assumed that the cell population in the target is of the order of 10^6^ cells in order to have a normalised slope of the TCP curve similar to that of gliomas.

## 3. Results and Discussion

**Figure 3:**
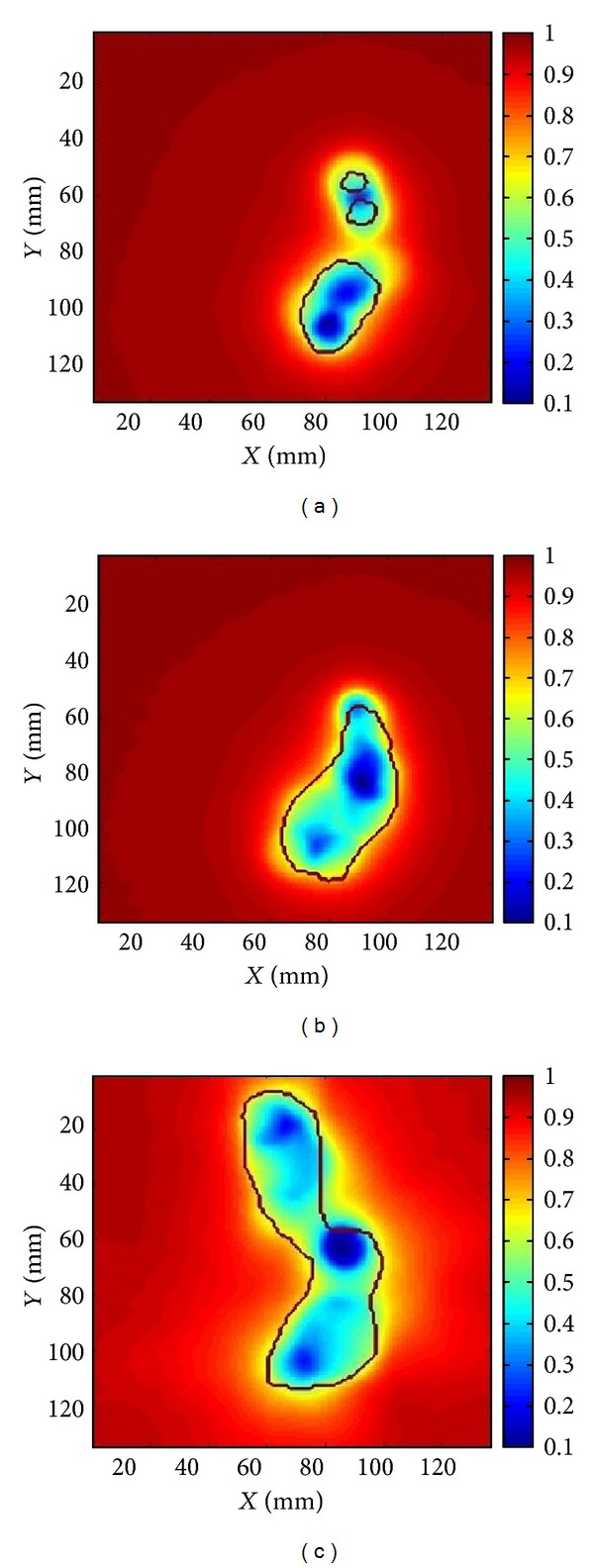
Distribution of cell survival in and around the target treated with stereotactic radiosurgery. The black contour is the planned 50% isodose. The colour scale indicates the surviving fraction of cells showing a very fast increase outside the area covered by the 50% isodose.

The distribution of cell survival in and around the target for each infiltration scenario considered in this study is shown in Figure 3.

The variation of cell survival within the target indicates that if the malignant cells are confined to the target, the high doses delivered to it are enough to sterilise all cells. However, if tumour cells may be found outside the delineated target, the steep dose falloff outside the target leads to lower cell killing in the regions where malignant cells may be infiltrating. This translates into very high TCP values for tumour cells confined to the treated volume and very poor outcome for infiltrations that are not accounted for during planning.

One might expect that the reduced cell kill might balance the decreased density of the infiltrating cells, but in reality the volume around the target in which infiltrating clonogenic cells might be encountered represents a volume equal to if not larger than the target (e.g., 10 mm infiltration distance around a 2 cm diameter spherical target represents a volume 7 times larger than the target itself). Hence, the relatively large volume represented by the margin effectively leads to rather high survival that is reflected in very low TCP. Thus, the calculated tumour control probability for the three cases showen in Figure 1 is dropping from 100% when no infiltration is assumed to 0% for the areas of infiltration illustrated in Figure 2 (Table 1).

These results support the idea that radiobiological outcome from SRS treatments could depend on the potential for infiltration of the cells in the treated tumour and the way this is taken into account at the stage of target delineation. Thus, the existence of the possible microscopic disease around the lesion visible on diagnostic images might effectively ruin the probability of controlling the tumour with SRS. This is important for many brain tumours that are candidates for SRS. Indeed, a study by Yamahara et al. [5] compared magnetic resonance (MR) images with pathological samples of glioblastoma multiforme (GBM) and showed that tumour cells may exist as far as 14 mm outside the tumour boundary determined on MR images. Similarly, Pirzkall et al. [8] verified the validity of magnetic resonance spectroscopy (MRSI) in defining the extent of glioma infiltration and suggested an addition of 2-3 cm margin to the gross tumour volume. Another study by the same group showed that the infiltration is heterogeneous and therefore a nonuniform margin might be needed [9].

The results in this study have been obtained under the assumption that cell density is constant within the delineated target. However, the delineated target shows heterogeneous uptake of contrast agents used to identify lesions in diagnostic images, which may also indicate heterogeneous cell density. Investigating the relationship between contrast uptake and cell density is however beyond the purpose of the present study that aimed to construct a radiobiological formalism for SRS evaluation taking into account possible infiltrations of malignant cells outside the target. Nevertheless, our results indicate that the existence of undetected tumour cells outside the volume receiving therapeutic doses might effectively lead to treatment failure as they could regrow the tumour following the treatment. It could be that, in some cases, when for example, some margins are included in the target, the doses outside it might be enough to sterilise the lower number of tumour cells infiltrated in the surrounding normal tissue. In others, however, the high gradients around the target might effectively prevent the sterilisation of the microscopic disease leading to a recurrence near the treated volume. From this perspective, further studies on the distribution of malignant cells in and around the lesions visible on diagnostic images are therefore warranted.

The simulations in this study have been performed with the LQ model. While the validity of the LQ model has been debated in recent years for SRS treatments employing large fractional doses delivered to the target [10, 11], it is important to recognise that the focus of this study has been on the effects in regions around the target that receive lower than therapeutically prescribed doses. For these doses, the validity of the LQ model is not a matter of debate and therefore the significance of the results cannot either be debated. They clearly indicate that if cell kill outside the target does not counterbalance the infiltrating cells, the outcome of the treatment might be poorer than expected.

The results of the present study therefore highlight the importance of target delineation and warrant further studies regarding patterns of failure for tumours treated with SRS and the relationship to the potential for infiltration of the treated tumour.

## 4. Conclusions

A radiobiological framework for the evaluation of treatment plans for invasive brain tumours was developed, taking into account the invasiveness of the tumour into the surrounding normal tissues. This offers a further criterion for the evaluation of stereotactic radiosurgery plans besides the conformity of therapeutic isodoses to the defined target and the gradients outside the target.

## Figures and Tables

**Table 1:** Tumour control probability for the three plans in Figure 1 and the corresponding scenario for the invasiveness of the astrocytoma clonogenic cells.

	TCP (%)		
	Case 1	Case 2	Case 3
*No infiltration* of clonogens outside the target	100	100	100
*Continuous decrease* of density of clonogens outside the target	0	0	0
*Heterogeneous decrease* of density of clonogens outside the target	0	0	0
